# Age-appropriate BMI cut-offs for malnutrition among older adults in India

**DOI:** 10.1038/s41598-024-63421-0

**Published:** 2024-07-02

**Authors:** Akancha Singh, Aparajita Chattopadhyay

**Affiliations:** 1 International Institute for Population Sciences, Mumbai, 400088 Maharashtra India; 2 Department of Population and Development, and Associate Head, Centre for Demography of Gender (CDG), International Institute for Population Sciences, Mumbai, 400088 Maharashtra India

**Keywords:** BMI, Older adults, CART analysis, Cut-off, Decision tree, India, Health care, Risk factors

## Abstract

With the increasing prevalence of obesity in India, body mass index (BMI) has garnered importance as a disease predictor. The current World Health Organization (WHO) body mass index (BMI) cut-offs may not accurately portray these health risks in older adults aged 60 years and above. This study aims to define age-appropriate cut-offs for older adults (60–74 years and 75 years and above) and compare the performance of these cut-offs with the WHO BMI cut-offs using cardio-metabolic conditions as outcomes. Using baseline data from the Longitudinal Ageing Study in India (LASI), classification and regression tree (CART) cross-sectional analysis was conducted to obtain age-appropriate BMI cut-offs based on cardio-metabolic conditions as outcomes. Logistic regression models were estimated to compare the association of the two sets of cut-offs with cardio-metabolic outcomes. The area under the receiver operating characteristic curve (AUC), sensitivity and specificity were estimated. Agreement with waist circumference, an alternate measure of adiposity, was conducted. For older adults aged 60–74 years and 75 years and above, the cut-off for underweight reduced from < 18.5 to < 17.4 and < 13.3 respectively. The thresholds for overweight and obese increased for older adults aged 60–74 years old from >  = 25 to > 28.8 and >  = 30 to > 33.7 respectively. For older adults aged 75 years and above, the thresholds decreased for both categories. The largest improvement in AUC was observed in older adults aged 75 years and above. The newly derived cut-offs also demonstrated higher sensitivity and specificity among all age-sex stratifications. There is a need to adopt greater rigidity in defining overweight/obesity among older adults aged 75 years and above, as opposed to older adults aged 60–74 years old among whom the thresholds need to be less conservative. Further stratification in the low risk category could also improve BMI classification among older adults. These age-specific thresholds may act as improved alternatives of the current WHO BMI thresholds and improve classification among older adults in India.

## Introduction

Body mass index (BMI) is an anthropometric measure comprising of an individual’s weight and height (BMI = weight (kg)/height^2^ (m))^1^. It is commonly used to measure nutrition status in populations. Owing to its ease of calculation and interpretation, it is used to guide many public policies and interventions^2^. BMI values were categorised into four groups by the World Health Organisation (WHO) in 1995, namely, underweight (BMI less than 18.5), normal (18.5 to less than 25), overweight (25 to less than 30), and obese (30 and above)^3,4^. These thresholds were selected based on a visual inspection of the association between BMI and mortality, and were later generalised to all ages^3,5,6^. Despite the universal usage of WHO BMI thresholds, there are certain drawbacks when these cut-offs are applied to older adults (aged 60 years and above). First, there is a change in body composition with age with respect to fat distribution^7–9^. Both cross-sectional^10–12^ and longitudinal^13–16^ have shown that there is an increase in intra-abdominal fat mass with age and this increase is more pronounced in women^7^. High levels of intra-abdominal fat mass can lead to insulin resistance, which, in turn, causes type-2 diabetes and cardiovascular diseases^17^. Along with the increase and redistribution of fat mass, there is a concomitant decrease in lean body mass with age, mostly owing to sarcopenia^13,14,17^. Since BMI does not take into account the amount of fat and muscle present and the location or type of fat, it does not accurately portray the health risks associated with adiposity and/or sarcopenia^18^. Second, BMI values also depend on precise measurements of height. In older adults, it is difficult to measure height precisely because there is a possibility of underestimation of height because of spinal discordances and/or an inability to stretch hips and knees^19^. Additionally, findings from several studies state that BMI might not accurately portray health risks in older adults. In adults considered overweight, mortality risk is often lower than their counterparts with normal BMI and this association is true for all ages and genders^4,20–22^.

Despite these limitation of BMI, studies suggest that BMI is well correlated with body fat, with correlations ranging from 0.72 to 0.86 and this correlation varies with age, sex and ethnicity^23,24^. Studies also suggest that the relationship of BMI and adiposity changes with age among adults and, thus, a one size fits all approach to BMI cut-offs may not be accurate^23,25–29^. Many studies have reiterated the need for devising age and sex-specific BMI cut-offs pertaining to certain health outcomes^2,3,18,30,31^. Although BMI performs better than other alternatives in measuring nutrition status, it is still crucial to establish context specific cut-offs. These will be helpful in singling out more vulnerable sections among older adults and guiding policy decisions.

The current WHO BMI thresholds were created using data from adults aged 18 years and above and, then generalising these cut-offs to all adults. This is perhaps not so robust because of the changes in body composition that occur with age. Thus, it is essential to revise BMI cut-offs, especially for older adults. Interestingly, there are more accurate body composition measures such as Dual-energy X-ray absorptiometry (DEXA) imaging. However, BMI remains the most inexpensive and easy measure and this makes it all the more important to rethink this critical programme measure. With the older adult population in India expected to become about 20% of the total population by 2050^32^ and the prevalence of both overweight and undernutrition affecting this population^33,34^, it is crucial to understand how current BMI thresholds will associate with their diagnosis and treatment and whether new BMI thresholds will offer an improvement over the previous one.

Despite the increasing importance of BMI as a disease predictor, there have been criticisms when applying the WHO cut-offs to the Asian population and attempts have been made to re-classify BMI as per the Asian population (underweight (< 18.5 kg/m^2^), normal weight (18.5–22.9 kg/m^2^), overweight (23–24.9 kg/m^2^), and obese (≥ 25 kg/m^2^)^6,35,36^. However, owing to the popularity and widespread usage of the WHO BMI cut-offs, the study chose to take them as the basis for comparison with the new cut-offs. While the association between high BMI/obesity and risk of diseases is well established^37–40^, there are conflicting findings when it comes to low BMI/underweight and cardiovascular diseases. Studies state that increased BMI is linked to increased mortality risk, but it remains unclear whether the risk increase with overweight^41^ or underweight also affects this risk^42,43^. Moreover, a J or U shaped relationship has been observed between BMI and both all-cause mortality and cardiovascular related mortality, with lowest mortality in the range of 25–30 kg/m^2^^44,45^. Therefore, it is important to see whether BMI at either extremes of the continuum is associated to CVDs and whether the new cut-offs offer improved diagnosis and classification.

Cardiovascular diseases (CVD) are commonly used to evaluate the health status in older adults. As individuals age, those who are overweight or obese are at a higher risk with respect to certain cardiovascular conditions such as hypertension and heart disease. These two conditions are closely linked with malnutrition among older adults^46–50^. CVDs are responsible for approximately one-third of global deaths and are the leading cause of death in India alone^51^. Moreover, there is a global rise in the prevalence of diabetes, with about 14.3% older adults reporting being diagnosed with diabetes^52^. Owing to the pervasiveness of these conditions, it will be crucial to see how new BMI thresholds perform with respect to these conditions. This paper aims to explore the relationship between BMI and health status to create novel age-appropriate BMI cut-offs that offer improved insights into health risks faced by older adults in India. The questions that we seek to answer are as follows: are our newly created BMI cut-offs more accurate than the traditional WHO cut-offs? Which of the two cut-offs perform better in relation to the selected disease outcomes among older adults in India?

## Data and methods

### Data

The data for this study was taken from the first wave of a prospective cohort study “Longitudinal Ageing Study of India (LASI)-Wave-1. This is a nationally representative survey of adults aged 45 and above across all states and union territories of India which collects information on disease, health and healthcare and socio and economic well-being of older adults. The data was collected between April 2017 and December 2018. The survey adopted a multistage stratified area probability cluster sampling design with stage stratification and 3–4 stages of sample selection. Face to face interviews were done using Computer Assisted Personal Interview (CAPI). The final sample size of the survey was 72,250 with individual and household response rates of 87.3 and 95.8 respectively. The dataset is available in public domain. More information about the methods and procedures for data collection can be found elsewhere^26^.

For this study, we first merged individual files and biomarker files using one-to-one matching to assess the information on anthropometry measures such as height and weight. We applied relevant sampling weights so that each state was represented in proportion to its population size. The hierarchical nature of data was taken into account using the *svyset* command in Stata and *india individual weight* variable in the LASI Individual data fil was used as weight for the purpose of the analysis. There were about 3413 individuals whose weight and height measurements were missing. These cases were dropped to obtain a representative sample of older adult Indian population aged 60 years and above. This dataset was then divided into two parts: the first one consisted data for older adults aged 60 to 74 years old (sample size- 22,348) and the second one consisted data for older adults aged 75 years and above (sample size- 5702). This division has been done on the basis of previous studies that have bifurcated data based on age: 60 (65)-74 as young old; 75–84 as middle-old and 85 and above as oldest-old^53–56^. This division is made on the basis of increasing global recognition that older adults are not a homogeneous group^57–59^ and conceptions of aging including biological, social, chronological and psychological differences^59^. Owing to sample size limitations, the division in this study has been made into only two age groups.

### Outcome variable

BMI was calculated by dividing weight in kilograms by height in metres squared. Height was measured in centimetres using a stadiometer, and weight was measured in kilograms using a Seca 803 digital weighing scale. BMI was used to assess nutrition status among the elderly and was categorised using World Health Organization (WHO) cutoffs- as < 18.5 kg/m^2^ (underweight), 18.5– 24.9 kg/m^2^ (normal weight), > 25.0 kg/m^2^ (overweight/obese)^27^. Hereafter, we refer to both overweight and obese individuals as overweight.

### Selection of health measures

#### Cardio-metabolic outcome

A list of chronic diseases were selected to assess the health status of individuals and the list was narrowed down to three diseases: (1) heart disease; (2) hypertension and (3) diabetes. The composite cardio-metabolic outcome was then formed based on these three variables. In LASI, the following questions were asked to collect data about the above-mentioned conditions:

Ever diagnosed with chronic heart diseases.

Ever diagnosed with hypertension.

Ever diagnosed with diabetes.

Less than 1% of the responses to the questions were answered as don’t know or were missing. A respondent was considered as having the composite cardio-metabolic outcome if they indicated ‘yes’ to any one of the above questions and not having the outcome otherwise.

### Statistical analysis

#### CART decision tree analysis

Age-stratified classification and regression tree (CART) analysis was conducted to determine appropriate BMI thresholds for older adults aged 60 years and above using cardio-metabolic outcomes as the health indicator. Logistic regression analysis was also conducted to assess the magnitude and direction of relationship between the WHO or the new BMI thresholds and health status. Receiver Operating Characteristic (ROC) curve was conducted to compare the performance of the newly derived thresholds from the WHO BMI cut points. We computed the area under the curve (AUC), sensitivity and specificity for both the newly derived cut-offs and the traditional WHO cut-offs.

The CART analysis is a non-parametric machine learning algorithm with high prediction performance. We chose this method, because unlike other decision tree analyses, CART does not necessitate predefining the relationship between the predictor variables and the variable of interest. This allowed us some flexibility in selecting health outcome measures. Additionally, we also did not need to predefine the number of cut-points for CART. CART algorithm works through recursive partitioning of a dataset to obtain subsets that are as pure as possible to a given target class. It constructs a binary decision tree by splitting a root node, which contains the whole data sample, into two child nodes, based on the target/dependent variable. The child nodes are further split in a binary fashion, and so forth until the tree reaches maximum depth. A sample binary decision tree, with labelled key features, is shown in Supplementary Fig. 1. The aim of a CART decision tree is to segment the data in a way that creates as close to pure terminal nodes as possible. A *pure* node consists of a node containing identical values of the target/dependent variable (i.e. cardio-metabolic outcome). CART analysis finds splits that maximize the homogeneity of the nodes in regards to this target variable. In order to do so, the CART algorithm uses a splitting criterion called the Gini impurity index. The impurity value is calculated by summing the probability (*pi*) of an item (*i)* being selected times the probability (Σ*pk* = *1-pi*) of a mistake in categorizing *i*. At a node *t*, the Gini impurity measure for CART algorithm is defined as:

$$i\left( t \right) \, = {\text{S}}i,j \, C \, \left( {i|j} \right)p\left( {i|t} \right)p(j|t)$$

where *C (i|j)* is the cost of misclassifying a class *j* case as a class *i*, and *p(i|t)/(j|t)* is the probability of a case in class *i/j* given that it falls in node.

*t*. When a node reaches purity, the Gini impurity value is zero (minimum). The CART algorithm therefore aims to minimize the impurity metric, as the objective of classification is ultimately to allocate participants in groups with minimal error and the least number of splits.

The CART decision tree analysis was conducted on the first wave of LASI data for older adults aged 60 years and above and the analysis was age-stratified by the following two age groups: 60–74, and 75 + years old. The analysis was age-stratified in order to capture the difference in BMI performance among the two groups of older adults, as it was expected that WHO-BMI groups would not classify older adults according to health risk as accurately.

All analyses were done using IBM SPSS Modeler (Version 18 for Windows).

#### Model validation

##### Split comprehensive data into training and testing data

The entire comprehensive dataset was split into training and testing subsets to avoid overfitting of the model. The training dataset alone was used to derive the BMI cut-offs using CART analysis. Cut-offs were obtained for both training and testing datasets and their performance was assesses. Typically, a training/testing split of 70%/30% or 80%/20% is sufficient, with the latter option suited well to larger datasets. We randomly selected 80% of the cases from the comprehensive dataset to generate the subset of training data. The remaining random 20% of cases were allotted to the testing data subset.

##### Logistic regression and ROC analysis

We constructed age-stratified logistic regression models to assess the strength and direction of association between BMI thresholds and cardio-metabolic outcome in both training and testing subsets. This was done to allow cross-validation of cut-off performance.

ROC analysis and area under the curve (AUC) was done to assess the diagnostic accuracy of the cardio-metabolic derived cut-offs. The AUC is a measure of diagnostic and/or predictive accuracy of the logistic regression model and assesses the model’s performance in distinguishing between positive and negative outcomes. It lies between 0 and 1, with higher values suggesting better predictive ability. Through our analysis, we expect to see an improvement in AUC with CART derived outcomes as compared to WHO cut-offs.

##### Sensitivity and specificity of CART derived thresholds

Sensitivity and specificity are measures used to interpret the clinical utility of a screening test. Sensitivity refers to the ability of a test to correctly identify those with the disease, while specificity is the ability to correctly identify those without disease. A test with high sensitivity has low specificity and vice versa.

We reported the sensitivity and specificity of both WHO BMI cut-offs and the CART derived thresholds to enable comparison. We also reported the AUC for both the cut-offs.

##### Construct validity: agreement with waist circumference (WC)

Waist circumference (WC) is a commonly used alternative to BMI for predicting disease risk among diverse populations as it is correlated highly with BMI^60^. The World Health Organization recommends the following WC cut-offs: > 94 cm (men)/80 cm (women), which corresponds to an increased risk of metabolic complications, and > 102 cm (men)/88 cm (women), which corresponds to a substantially increased risk. For the purposes of this analysis, WC was thus categorized as such: Low risk (≤ 94 cm (men) or 80 cm (women)), Increased Risk (> 94 cm (men)/80 cm (women) to ≤ 102 cm (men)/88 cm (women)), and Substantially Increased Risk (> 102 cm (men)/88 cm (women)). The agreement statistic reported for this analysis was the weighted Cohen’s kappa, which takes into account the ordering of the categories used and assigns a weighting to the degree of disagreement accordingly. For this analysis, linear weighted kappa was selected, as the linear weight should be used when the difference between the first two categories (i.e. Low Risk and Increased Risk) is equally as important as the difference between the last two (i.e. Increased Risk and Substantially Increased Risk). Kappa confidence intervals and observed agreement (%) were also reported. It is expected that the CART-derived BMI cut-offs demonstrate higher agreement values with WC, when compared to WHO-BMI cut-offs.

## Results

### Findings from CART analysis with cardio-metabolic (CM) outcomes

The age-stratified CART decision trees were presented in Supplementary Figs. 2 and 3. New BMI groupings were determined from the terminal nodes of the decision trees, with the left-most terminal node on a tree representing the first BMI grouping. For example, Supplementary Fig. 2 presents the BMI thresholds for CM outcomes derived for the age group 60–74. On this tree, Node 7 was the first terminal node and created the BMI grouping of ≤ 17.4. The second BMI grouping was derived from the next terminal node to its right, Node 15, which represented a BMI range of 17.4 to 19.3. The former threshold was derived from Node 8, which is the parent node to Node 15. Following this, the third BMI grouping was derived from terminal Node 16. The BMI range represented by Node 9 was from 19.3 to 19.9 (threshold taken from parent Node 3). This method of determining cut-points followed until the right-most, final terminal node in the tree (Node 14). The final grouping represented by Node 14 was > 33.7. This method of deriving BMI groupings from decision trees was followed for all subsequent trees.

Similarly, Supplementary Fig. 3 presented the BMI thresholds for CM outcomes for older adults aged 75 and above. For this age group, Node 7 was the first terminal node which yielded the first cut-off of ≤ 13.3. The second threshold was > 13.3 to ≤ 17.4. This was obtained from the next terminal node (Node 8) to the right of Node 7. The latter part of the threshold was obtained from Node 3 (parent node to Node 8). Moving further rightwards, the next threshold was < 17.4 to ≤ 20.0. Like the previous cut-offs, this was obtained from the third terminal node (Node 9), with the former part of the threshold taken from Node 4 (parent node to Node 9). As stated above, this method was followed until all the terminal nodes were considered.

These raw BMI groupings were then collapsed further in order to: (a) maintain a similar number of categories as the WHO-BMI cut-offs (N = 6), (b) retain clinical relevance (i.e. avoiding BMI ranges based on decimal place differences), and (c) maintain an increasing gradient of disease risk percentages. Multiple versions of the collapsed groupings were created, and their performance tested, in order to select the final version that most improved classification. The raw and final collapsed groupings, with disease frequencies within categories, were presented (Tables 1 and 2 in the Supplementary file).

**Table 1:** New BMI Thresholds Derived by CART Analysis with CM Outcomes (“BMI-CM-Risk Groups”).

Group name	60 to 74 years old	75 + years old	Corresponding WHO group
Increased risk (−)^1^	< = 17.4	< = 13.3	Underweight (< 18.5)
Low risk 1	> 17.4 to < = 19.9	> 13.3 to < = 20.0	Normal (> = 18.5 to < 25.0)
Low risk 2	> 19.9 to < = 22.9	> 20.0 to < = 21.5	
Low risk 3	> 22.9 to < = 28.8	> 21.5 to < = 22.8	
Increased risk ( +)^2^	> 28.8 to < = 33.7	> 22.8 to < = 28.7	Overweight (> = 25.0 to < 30.0
Substantially Increased risk	> 33.7	> 28.7	Obese (> = 30.0)

^1^Increased risk (−) corresponds to an increased health risk due to lower BMI.

^2^Increased risk ( +) corresponds to an increased health risk due to higher BMI.

**Figure 1:**
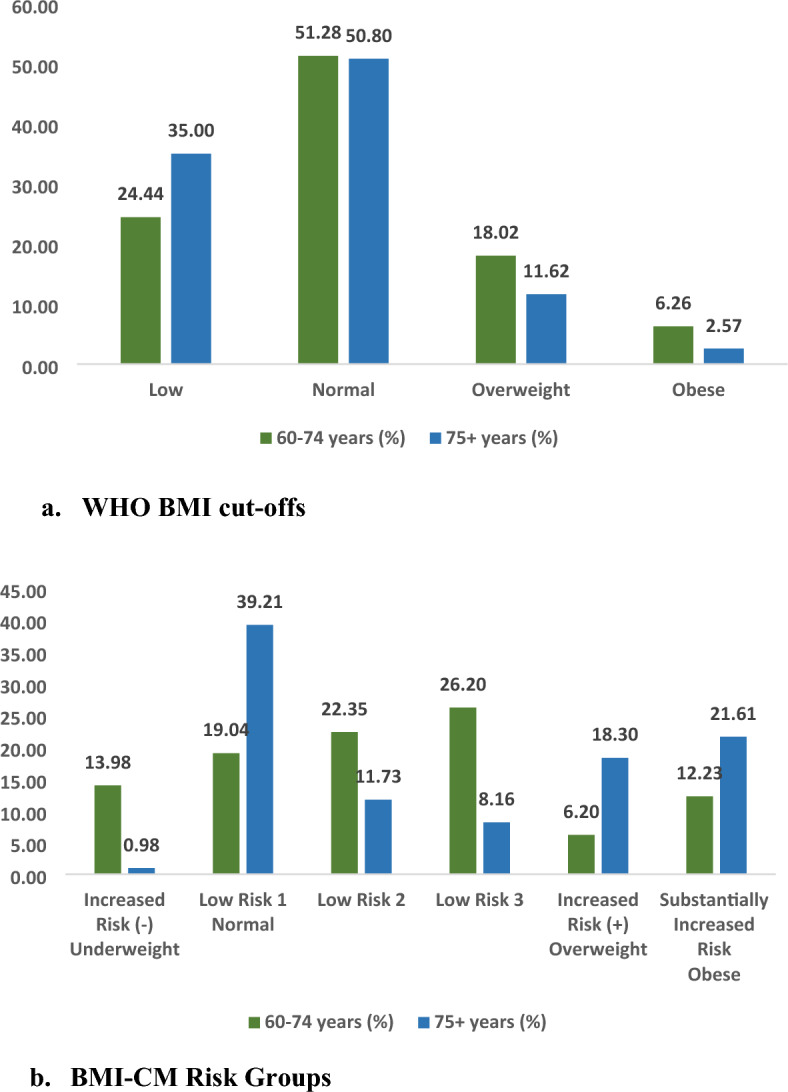
Distribution of older adult population across BMI categories, (**a**) WHO BMI cut-offs. (**b**) BMI-CM risk groups. Figure 1 (**a**) shows the distribution of older adults aged 60–74 years and 75 years across different WHO BMI cut-offs. In the age group 60–74, 24.4%, 51.3%, 18% and 6.3% older adults were underweight, in the normal BMI range, overweight and obese respectively. In the age group 75 years and above, 35%, 50.8%, 11.6% and 2.6% older adults were underweight, in the normal BMI range, overweight and obese respectively. Figure 1 (**b**) shows the distribution of older adults aged 60–74 years and 75 years across different BMI-CM risk groups. In the age group 60–74, 14% older adults belonged to the Increased risk (−) category. 39.2%, 11.7% and 8.2% older adults belonged to the Low risk 1, Low risk 2 and Low risk 3 categories respectively. 18.3% older adults belonged to the Increased risk ( +) category and 21.6% older adults belonged to the Substantially increased risk category. In the age group 75 years and above, 1% older adults belonged to the Increased risk (-) category. 19%, 22.4% and 26.2% older adults belonged to the Low risk 1, Low risk 2 and Low risk 3 categories respectively. 6.2% older adults belonged to the Increased risk ( +) category and 12.2% older adults belonged to the Substantially increased risk category.

**Table 2:** Logistic regression with WHO-BMI cut-offs as predictor variables.

Category	Unadjusted	Sex adjusted
	Odds Ratio (95% CI)	Odds Ratio (95% CI)
60–74 years old (Reference: Normal)		
Underweight	0.86** (0.17, 0.99)	0.93 (0.54, 2.11)
Overweight	1.92* (1.01, 2.84)	1.55* (1.23, 2.39)
Obese Class 1	2.11*** (1.23, 2.90)	2.92** (1.83, 3.67)
Obese Class 2	3.27* (2.01, 4.23)	3.02*** (2.32, 4.80)
Obese Class 3	3.99* (2.54, 4.97)	4.11** (2.98, 5.13)
75 + years old (Reference: Normal)		
Underweight	0.43* (0.21, 0.92)	0.81** (0.19, 0.99)
Overweight	1.21** (1.01, 3.11)	1.92** (1.11, 3.23)
Obese Class 1	1.98* (1.30, 3.91)	2.11*** (1.62, 3.01)
Obese Class 2	3.23*** (2.59, 4.12)	3.45* (3.11, 4.01)
Obese Class 3	3.82*** (3.11, 5.23)	4.01*** (2.99, 5.08)

*p < 0.01, **p < 0.05, ***p < 0.10.

Table 1 presented the final age-stratified cardio-metabolic-BMI cut-offs (henceforth referred to as “BMI-CM-Risk groups”) with comparisons to the WHO-BMI groups. These grouping were used to create six final BMI-CM risk groups, derived directly from decision trees.

Within the BMI-CM-Risk groupings, we gave new group names to focus on the pattern of disease risk (Table 3) in line with the research of Javed et al.^18^. The grouping comparable to “Underweight” was renamed to “Increased Risk (−)”, “Normal” to “Low Risk”, “Overweight” to “Increased Risk ( +)”, and “Obese” to “Substantially Increased Risk”. Compared to WHO-BMI thresholds, the cut-offs for “Increased Risk (−)” groups decreased slightly for older adults aged 60 to 74 (< = 17.4 vs. WHO threshold of < 18.5). For older adults aged 60 to 74, the normal range also changed a bit (> 17.4 to <  = 28.8 vs WHO threshold of >  = 18.5 to < 25.0). In the new BMI groups, the traditional “Normal” category was segmented into 3 levels of “Low Risk”. Similarly, the ‘Increased Risk ( +)” threshold changed (> 28.8 to <  = 33.7 vs WHO threshold of >  = 25 to < 30). This was also true for the “Substantially Increased Risk” category.

**Table 3:** Logistic regression with BMI-CM Risk groups as predictor variables.

Category	Unadjusted	Sex adjusted
	Odds Ratio (95% CI)	Odds Ratio (95% CI)
60–74 years old (Reference: Low Risk 1)		
Increased Risk (−)	1.01 (0.43, 2.11)	0.80 (0.38, 1.90)
Low Risk 2	1.28** (1.11, 2.93)	1.55** (1.08, 3.23)
Low Risk 3	1.56*** (1.21, 3.19)	1.62*** (1.20, 3.11)
Increased Risk ( +)	3.34** (2.87, 4.01)	3.99*** (2.93, 4.81)
Substantially Increased Risk	4.12*** (3.59, 5.51)	5.61** (2.04, 6.33)
75 + years old (Reference: Low Risk 1)		
Increased Risk (−)	1.82* (1.11, 2.11)	1.39*** (1.19, 2.37)
Low Risk 2	1.11*** (1.02, 3.25)	1.08 (0.92, 2.11)
Low Risk 3	1.23*** (1.05, 2.99)	1.33*** (1.20, 2.76)
Increased Risk ( +)	2.96** (1.70, 4.55)	3.62* (2.34, 4.26)
Substantially Increased Risk	3.96** (1.84, 4.78)	4.80* (2.66, 5.19)

*p < 0.01, **p < 0.05, ***p < 0.10.

For older adults aged 75 and above, the “Increased Risk (−)” threshold changed (< = 13.3 vs WHO threshold of <  = 18.5). The ‘Low Risk” category changed as well (> 13.3 to <  = 22.8 vs WHO threshold of <  = 18.5 to < 25). This was also true for ‘Increased Risk ( +)” and “Substantially Increased Risk” category.

### Results from logistic regression analyses

Table 2 presented results from logistic regression analysis where WHO BMI threshold is the main predictor variable. We ran two models: the first one was unadjusted and the second one was adjusted for sex to see if that accounted for the association between BMI thresholds and cardio-metabolic status. The first part shows results for older adults aged 60 to 74 years old. Older adults who were underweight were 0.14 (95% CI 0.17, 0.99) times less likely to have CM conditions than their counterparts in the normal BMI category. Those in the overweight category were 0.92 times (95% CI 1.01, 2.84) more likely to have CM conditions. This association was true for all three classes of obesity as well.

In the sex adjusted model, those who were overweight were 0.55 times (95% CI 1.23, 2.39) more likely to have CM condition. Older adults belonging to obese class 1, obese class 2 and obese class 3 were 1.92 (95% CI 1.83, 3.67), 2.02 (95% CI: 2.32, 4.80) and 3.11 (95% CI: 2.98, 5.13) times more likely to have CM conditions respectively.

In the unadjusted model, older adults aged 75 and above who were underweight were 0.57 times (95% CI 0.21, 0.92) less likely to have CM conditions than their counterparts in the normal BMI category. Overweight older adults were 0.21 times more likely (95% CI 1.01, 3.11) to have CM conditions than their counterparts. Older adults belonging to obese class 1, 2 and 3 were 0.98 (95% CI 1.30, 3.91), 2.23 (95% CI 2.59, 4.12) and 2.82 (95% CI 3.11, 5.23) times more likely respectively to have CM conditions than their counterparts in the normal BMI category.

In the sex adjusted model, underweight older adults were 0.19 times (95% CI 0.19, 0.99) less likely to have CM conditions than their counterparts. Those belonging to obese class 1, 2 and 3 category were 1.11 (95% CI 1.62, 3.01), 2.45 (95% CI 3.11, 4.01) and 3.01 (95% CI 2.99, 5.08) times more likely respectively to have CM conditions than their counterparts in the normal weight category.

Table 3 presented results from logistic regression analysis where BMI-CM risk groups were the main predictor variable. The first part shows results for older adults aged 60 to 74 years old. In the unadjusted model, older adults belonging to the low risk 2 and low risk 3 category were 0.28 times (95% CI 1.11, 2.93) and 0.56 times (95% CI 1.21, 3.19) more likely respectively to have CM conditions than their counterparts in the low risk 1 category. Those who belonged to the increased risk ( +) category were 2.34 times (95% CI 2.87, 4.01) more likely to have CM conditions and those belonging to the substantially increased risk category were 3.12 times (95% CI 3.59, 5.51) more likely to have CM conditions.

In the sex adjusted model, older adults belonging to the low risk 2 and low risk 3 categories were 0.55 (95% CI 0.38, 1.90) and 0.62 (95% CI 1.20, 3.11) times more likely respectively to have CM conditions than their counterparts in the low risk 1 category. Older adults in the increased risk ( +) and substantially increased risk category were 2.99 (95% CI 2.93, 4.81) and 4.61 (95% CI 2.04, 6.33) times more likely respectively to have CM conditions than their counterparts.

The magnitude of odds ratios of older adults aged 75 and above was along similar lines like their counterparts aged 60 to 74.

We also obtained the age-stratified AUCs of BMI-CM risk groups against the WHO-BMI cut-offs. Table 4 showed the age-stratified AUCs for the training dataset. The AUC of WHO-BMI cut-offs for CM outcomes among older adults aged 60–74 years old was 0.66 as against the AUC of 0.69 for BMI-CM risk groups. This marked an improvement of 0.03 of BMI-CM risk groups over the WHO-BMI cut-offs. For older adults aged 75 and above, the AUC of WHO-BMI cut-offs for CM outcomes was 0.64 as against the AUC of 0.68 for BMI-CM risk groups, demonstrating an improvement of 0.04.

**Table 4:** Age-stratified AUCs of logistic regression models in training dataset.

BMI Cut-offs	Outcome	Stratification	AUC	Improvement from WHO-BMI Cut- Offs
WHO-BMI cut-offs	CM Outcome	60–74 years old	0.66	–
		75 + years old	0.64	–
BMI-CM-Risk Cut-offs	CM Outcome	60–74 years old	0.69	**0.03**
		75 + years old	0.68	**0.04**

Bolded values indicate improvement in AUC from WHO-BMI cut-offs. AUCs are derived from unadjusted models.

### Sensitivity, specificity and AUCs of BMI cut-offs

Table 5 showed the results of sensitivity, specificity and AUCs of both BMI cut-offs in the training dataset. This analysis was stratified by age and sex to see whether the proposed cut-offs demonstrated an improvement over BMI cut-offs for different sub-sections of the population as well. In males aged 60–74 years old, the sensitivity and specificity of WHO-BMI cut-offs were 66.0 and 33.5 respectively. The corresponding values for BMI-CM risk groups were 66.2 and 33.8 respectively, demonstrating an improvement of 0.02 and 0.03 in the sensitivity and specificity respectively. In males aged 75 years and above, BMI-CM risk groups recorded an improvement of 2.8 in sensitivity over WHO BMI cut-offs and the AUC was also 0.06 points higher for the former. Among women aged 60–74 years old and 75 years and above, the specificity of BMI-CM risk groups was 7.4 and 7.2 points higher respectively than WHO BMI cut-offs. The AUCs in both the age groups among were also 0.03 and 0.07 points higher respectively for BMI-CM risk groups than WHO BMI cut-offs.

**Table 5:** Sensitivity, Specificity, and AUCs of BMI cut-offs in Training Dataset.

		WHO-BMI Cut-offs			BMI-CM-Risk Cut-Offs		
		Sensitivity	Specificity	AUC	Sensitivity	Specificity	AUC
Men	60–74 years old	66.0	33.5	0.58	**66.2**	**33.8**	**0.69**
	75 + years old	62.3	37.7	0.62	**65.1**	**44.9**	**0.68**
Women	60–74 years old	65.4	30.8	0.66	63.1	**38.2**	**0.69**
	75 + years old	63.3	32.5	0.64	60.6	**39.7**	**0.71**

Bolded values indicate improvements in sensitivity, specificity, or AUCs from WHO-BMI cut-offs.

### Construct validity: agreement with waist circumference

For this purpose, BMI-CM risk groups were re-categorized into three groups: low risk, increased risk and substantially increased risk. Table 6 showed the weighted kappa values and their 95% confidence intervals (CIs). The kappa values among males aged 60–74 years old and 75 years and above were 0.72 and 0.66 respectively. In both the categories, the kappa value for BMI-CM risk groups was higher than that of WHO-BMI cut-offs. For males aged 60–74 years old, the increase was of 0.04 points and the corresponding increase was 0.11 points for males aged 75 years and above. For females aged 60–74 years old, the kappa value for WHO-BMI groups was higher than that of BMI-CM risk groups. However, for females aged 75 years and above, the kappa value of BMI-CM risk groups was 0.04 points higher than that of WHO-BMI cut-offs.

**Table 6:** Agreement Statistics between BMI Cut-Offs and Waist Circumference in Comprehensive Dataset.

Stratification	Waist circumference agreement with	Linear weighted kappa	95% confidence interval	
Men: 60–74 years old	WHO-BMI Cut-Offs	0.68	0.60	0.72
	60–74 Years Old BMI-CM-Risk Cut-Offs	0.72*	0.66	0.79
Men: 75 + years old	WHO-BMI Cut-Offs	0.55	0.48	0.60
	75 + Years Old BMI-CM-Risk Cut-Offs	0.66*	0.59	0.71
Women: 60–74 years old	WHO-BMI Cut-Offs	0.62	0.57	0.71
	60–74 Years Old BMI-CM-Risk Cut-Offs	0.59	0.55	0.69
Women: 75 + years old	WHO-BMI Cut-Offs	0.71	0.63	0.80
	75 + Years Old BMI-CM-Risk Cut-Offs	0.75*	0.70	0.82

*Improvement from WHO-BMI cut-offs.

## Discussion

This study used classification and regression tree (CART) analysis to define age-appropriate BMI thresholds for older adults in India. These thresholds, called BMI-CM risk groups, were created with respect to cardio-metabolic (CM) conditions (heart diseases, hypertension and diabetes). Our findings suggested that BMI-CM risk groups offered improved classification of older adults with respect to CM conditions as compared to WHO-BMI cut-offs. The BMI-CM risk groups demonstrated improvements in AUCs as compared to WHO-BMI cut-offs and these improvements were maximum for men aged 60–74 years old in the training dataset and women aged 60–74 years old in the testing dataset. The BMI-CM risk groups also showed higher agreement with waist circumference for most age-sex stratifications.

The ‘Low Risk 1’ groups served as the lowest level of health risk and, thus, was taken as the reference category in the regression analyses. Older adults belonging to all the other categories had higher odds of having CM conditions and this was true for both the age groups, namely, 60–74 years and 75 years and above and also for both unadjusted and unadjusted models. For both age groups, the odds of having CM conditions was higher in ‘Low Risk 2’ and ‘Low Risk 3’ categories as compared to the ‘Low Risk 1’ category. This grouping represented low health risks and, hence, this finding could be misconstrued as concerning. However, studies suggest that even people with traditional normal BMI have certain degree of health risks and this is especially true for people at the upper end of this BMI range^18^. In the LASI data, 34.8% and 44.6% of all normal BMI older adults aged 60–74 years and 75 years and above had one or more CM conditions of interest respectively. It is important to note that the odds of having CM conditions among ‘Low Risk Groups’ 2 and 3 were still lower than that of the traditional overweight and obese groups, suggesting that these groups were not a major cause of concern.

Our findings based on BMI-CM Risk groups suggested a decrease in the threshold for underweight, but an increase in the threshold for overweight among older adults aged 60–74. Among older adults aged 75 years and above, the BMI-CM risk groups suggested a decrease in the threshold for all three categories, namely, underweight, overweight and obesity. The Increased Risk (−) category corresponds to the WHO category of underweight. This is an important category because there are many studies that discuss the association of overweight and obesity with CVDs. However, studies linking underweight with CVDs, although rare, have also been coming up^45,61–63^. Additionally, a U-shaped relationship between BMI and mortality also establish the importance of this category^21,23,62,64,65^.

The latter finding has been established in literature stating that a decreased threshold for obesity could improve diagnosis chances in the oldest-old, especially women^18^. Other studies suggested that an obesity cut-off of 30 and above was inappropriately high for older adult women^66^. Post menopause, women experience increases in body fat and a decrease in skeletal mass, thus, affecting the relationship between BMI and body fat^31,67^. These changes could be one of the explanations behind lower cut-offs in the ‘Increased Risk ( +)’ and ‘Substantially Increased Risk’ categories in older adults aged 75 and above. Findings from this study, therefore, suggest that there is a need to adopt greater rigidity in defining overweight/obesity among older adults aged 75 years and above, as opposed to older adults aged 60–74 years old among whom the thresholds need to be less conservative. Additionally, further stratification in the low risk category could also improve BMI classification among older adults.

The ROC analysis revealed that the BMI-CM risk groups showed improvements in AUC over WHO BMI thresholds. While BMI-CM risk groups showed modest improvements in sensitivity, they recorded considerable increases in specificity across all age groups and for both the sexes. Greater improvements in AUCs for men and women aged 60–74 years old suggest that there is a need for age-specific cut-offs pertaining to BMI in India. Sensitivity and specificity are important markers of any screening test and provide clinical interpretability^68^. While the ideal screening test would be both highly sensitive and specific, some trade-offs may be worthwhile depending on the use of the test. For certain tests, specificity may be of greater importance^69^. For the purpose of this study, it is important to screen individuals without the disease condition as this will improve the classification of our thresholds. There are pertinent clinical implications of the above mentioned findings. Our BMI-CM age-stratified risk groups offer improvements in classification as far as CM conditions are concerned. The age-specific nature of these cut-offs will also provide with tailored cut-off points along the aging continuum. This is very important as there are considerable changes in body composition with increase in age^70^ and, hence, using the same cut-off for the entire age group may be factually incorrect.

The discoveries will significantly impact the perceptions of body image and mental well-being among older adults. The categorization names established for BMI-CM-Risk categories are crafted to correspond with disease risk levels rather than using terms like "normal" or "overweight." Employing language such as "normal" or other variations of BMI classifications like "desirable" can be problematic, potentially fostering a negative self-perception in individuals deviating from this supposed ideal^71,72^. From a clinical perspective, conveying the health risk associated with specific BMI ranges is a more objective approach. The current practice of using labels that may carry marginalizing or negative connotations could be detrimental. The stigma attached to being labeled as “overweight” or “obese” can result in individuals internalizing anti-fat biases and feeling dissatisfied with their bodies^73–75^. Moreover, these biases can lead to discrimination and adverse psychological impacts on mental health, self-esteem, and body satisfaction. These societal prejudices regarding weight are pervasive in media and are even prevalent among the attitudes of medical professionals and researchers^76,77^.

The main strength of the study is that it offers a new approach and is one of the first studies to re-categorize the WHO BMI groupings with respect to disease risks in the Indian older adult population. It is expected that by 2050, about 20 percent of India’s population will be constituted by older adults^78^. This makes it more important to improve the performance of easy to use screening tools like BMI. The BMI-CM risk thresholds, as proposed by this study, allows for more accurate screening of health risks among older adults, while also maintaining the ease of use and interpretation of BMI. While there are certain alternative measures of weight and adiposity, they all have several limitations. Measures such as dual energy X-ray absorptiometry (DEXA) imaging and underwater weighting are more accurate measures of body fatness, but they are expensive and inaccessible to most^67^. Other less expensive methods are bioelectrical impedance analysis (BIA) and skin fold thickness but they are limited in accuracy and by skills of the examiner^79–81^. Thus, revising current BMI thresholds and tailoring them to populations of interest is a worthwhile endeavour.

There are certain limitations to the current study. While our BMI-CM risk groups are age-specific, they are not sex-specific. This was because further stratifying would have resulted in small sample sizes and this would have produced inconsistent results. This is a limitation because capturing sex based differentials is a crucial aspect in capturing the relationship between BMI and health risks and this is especially true for menopausal women^66^. However, our age and sex stratified analyses show that the BMI-CM risk groups still offer improvements over the WHO BMI cut-offs. Second, we used non-parametric methods to obtain the decision trees. Research suggests that no-parametric decision trees tend to over-fit data and, hence, they make the application of findings to independent datasets difficult^82,83^. To deal with potential over-fitting, we partitioned our data into training and testing subsets and used stopping rules. In addition to the above, decision trees have high variance across samples^84^, meaning that results can vary greatly even if smallest changes are made to the sample. This happens due to the nature of the splitting process wherein slight changes at early splits could impact subsequent child nodes^83^. Moreover, since the LASI data is cross-sectional, we could not explore the performance of our proposed BMI cut-offs over time.

While the proposed age-specific cut-offs demonstrate improvements in classifying older adults in LASI data, further research is needed to ascertain their functionalities in other populations and ethnicities and in the context of other health outcomes. Additionally, subject to the availability of longitudinal data, BMI-mortality relationship could be explored using these cut-offs. Longitudinal data could also be used for understanding how changes in BMI and the transition between these revised groupings would impact health outcomes. Further studies could also consider the testing how self-reported BMI data functions with respect to these groupings. Lastly, while further exploration of these BMI cut-offs is important, it is also crucial to bolster the development of alternate improved indicators of nutrition, which maintain the accessibility of BMI but also include the accuracy of its alternatives.

## Conclusion

This study is a new attempt at defining age-appropriate BMI cut-offs for older adults. The cut-offs for older adults aged 60–74 years are: <  = 17.4 (underweight), > 17.4 to <  = 28.8 (normal), > 28.8 to <  = 33.7 (overweight) and > 33.7 (obese). For older adults aged 75 years and above, the cut-offs are <  = 13.3 (underweight), > 13.3 to <  = 22.8 (normal), > 22.8 to <  = 28.7 (overweight) and > 28.7 (obese). Thus, the current study proposes lower underweight threshold and higher overweight and obesity thresholds for older adults aged 60–74 years old and higher underweight and overweight/obesity thresholds for older adults aged 75 years and above. These thresholds performed better than WHO-BMI cut-offs for both the age groups- 60–74 years old and 75 years and above in terms of them being more strongly associated with cardio-metabolic outcomes as shown in logistic regressions and showing improved sensitivity, specificity and AUC as compared to the WHO cut-offs. These findings strengthen the need for age specific cut-offs among older adults. Due to changes in body composition with age and redistribution of body fat, the current BMI thresholds may not accurately reflect health risks in older age in India. These age-specific thresholds may act as improved alternatives of the current WHO BMI thresholds and improve classification among older adults in India.

### Supplementary Information

Supplementary Information.

## Data Availability

The data is available in public domain and is accessible on request from LASI—Data | International Institute for Population Sciences (IIPS) (iipsindia.ac.in).

## References

[1] BMI Calculator Harvard Health. Available at: https://www.health.harvard.edu/diet-and-weight-loss/bmi-calculator. Accessed 28 June 2023, (2015).

[2] Evans B, Colls R (2009). Measuring fatness, governing bodies: The spatialities of the body mass index (BMI) in anti-obesity politics. Antipode.

[3] Physical status : The use of and interpretation of anthropometry, report of a WHO expert committee Available at: https://www.who.int/publications-detail-redirect/9241208546. Accessed 18 October (2022). 8594834

[4] Nuttall FQ (2015). Body mass index: Obesity, bmi, and health: A critical review. Nutr. Today.

[5] WHO Consultation on Obesity (1999: Geneva S, Organization WH Obesity : preventing and managing the global epidemic : report of a WHO consultation. World Health Organization Available at: https://apps.who.int/iris/handle/10665/42330. Accessed 28 June 2023. (2000). 11234459

[6] WHO Expert Consultation (2004). Appropriate body-mass index for Asian populations and its implications for policy and intervention strategies. Lancet.

[7] Kuk JL, Saunders TJ, Davidson LE, Ross R (2009). Age-related changes in total and regional fat distribution. Ageing Res. Rev..

[8] Zamboni M, Armellini F, Harris T, Turcato E, Micciolo R, Bergamo-Andreis IA, Bosello O (1997). Effects of age on body fat distribution and cardiovascular risk factors in women. Am. J. Clin. Nutr..

[9] Gallagher D, Ruts E, Visser M, Heshka S, Baumgartner RN, Wang J, Pierson RN, Pi-Sunyer FX, Heymsfield SB (2000). Weight stability masks sarcopenia in elderly men and women. Am. J. Physiol. Endocrinol. Metab..

[10] Shimokata H, Tobin JD, Muller DC, Elahi D, Coon PJ, Andres R (1989). Studies in the distribution of body fat: I. Effects of age, sex, and obesity. J. Gerontol..

[11] Teh BH, Pan W, Chen CJ The reallocation of body fat toward the abdomen persists to very old age, while body mass index declines after middle age in Chinese. International journal of obesity and related metabolic disorders : journal of the International Association for the Study of Obesity. Available at: https://www.semanticscholar.org/paper/The-reallocation-of-body-fat-toward-the-abdomen-to-Teh-Pan/a44a596b36bcf0934f5f87b5bd48bbfdb54f0cf1. Accessed 28 June 2023. (1996). 8817363

[12] Ito H, Ohshima A, Ohto N, Ogasawara M, Tsuzuki M, Takao K, Hijii C, Tanaka H, Nishioka K (2001). Relation between body composition and age in healthy Japanese subjects. Eur. J. Clin. Nutr..

[13] Noppa H, Andersson M, Bengtsson C, Bruce A, Isaksson B (1980). Longitudinal studies of anthropometric data and body composition. The population study of women in Götenberg Sweden. Am. J. Clin. Nutr..

[14] Carmelli D, McElroy MR, Rosenman RH (1991). Longitudinal changes in fat distribution in the Western Collaborative Group Study: a 23-year follow-up. Int. J. Obes..

[15] Zamboni M, Zoico E, Scartezzini T, Mazzali G, Tosoni P, Zivelonghi A, Gallagher D, De Pergola G, Di Francesco V, Bosello O (2003). Body composition changes in stable-weight elderly subjects: the effect of sex. Aging Clin. Exp. Res..

[16] Hughes VA, Roubenoff R, Wood M, Frontera WR, Evans WJ, Fiatarone Singh MA (2004). Anthropometric assessment of 10-y changes in body composition in the elderly. Am. J. Clin. Nutr..

[17] Beaufrère B, Morio B (2000). Fat and protein redistribution with aging: Metabolic considerations. Eur. J. Clin. Nutr..

[18] Javed AA, Ma J, Anderson LN, Mayhew AJ, So HY, Griffith LE, Gilsing A, Raina P (2022). Age-appropriate BMI cut-points for cardiometabolic health risk: A cross-sectional analysis of the Canadian longitudinal study on aging. Int. J. Obes.

[19] Sorkin JD, Muller DC, Andres R (1999). Longitudinal change in the heights of men and women: consequential effects on body mass index. Epidemiol. Rev..

[20] Pischon T, Boeing H, Hoffmann K (2008). General and abdominal adiposity and risk of death in Europe. N. Engl. J. Med..

[21] Adams KF, Schatzkin A, Harris TB, Kipnis V, Mouw T, Ballard-Barbash R, Hollenbeck A, Leitzmann MF (2006). Overweight, obesity, and mortality in a large prospective cohort of persons 50 to 71 years old. N. Engl. J. Med..

[22] Zhu Y, Wang Q, Pang G, Lin L, Origasa H, Wang Y, Di J, Shi M, Fan C, Shi H (2015). Association between Body Mass Index and Health-Related Quality of Life: The ‘Obesity Paradox’ in 21,218 Adults of the Chinese general population. PLOS One.

[23] Flegal KM, Shepherd JA, Looker AC, Graubard BI, Borrud LG, Ogden CL, Harris TB, Everhart JE, Schenker N (2009). Comparisons of percentage body fat, body mass index, waist circumference, and waist-stature ratio in adults. Am. J. Clin. Nutr..

[24] Misra P, Singh AK, Archana S, Lohiya A, Kant S (2019). Relationship between body mass index and percentage of body fat, estimated by bio-electrical impedance among adult females in a rural community of North India: A cross-sectional study. J. Postgrad. Med..

[25] Meeuwsen S, Horgan GW, Elia M (2010). The relationship between BMI and percent body fat, measured by bioelectrical impedance, in a large adult sample is curvilinear and influenced by age and sex. Clin. Nutr..

[26] Gallagher D, Visser M, Sepúlveda D, Pierson RN, Harris T, Heymsfield SB (1996). How useful is body mass index for comparison of body fatness across age, sex, and ethnic groups?. Am. J. Epidemiol..

[27] Kim SG, Dong KOK, Hwang IC, Suh HS, Kay S, Caterson I, Kim KK (2015). Relationship between indices of obesity obtained by anthropometry and dual-energy X-ray absorptiometry: The Fourth and fifth Korea national health and nutrition examination survey (KNHANES IV and V, 2008–2011). Obes. Res. Clin. Pract..

[28] Morabia A, Ross A, Curtin F, Pichard C, Slosman DO (1999). Relation of BMI to a dual-energy X-ray absorptiometry measure of fatness. Br. J. Nutr..

[29] Batsis JA, Mackenzie TA, Lopez-Jimenez F, Bartels SJ (2015). Sarcopenia, sarcopenic obesity, and functional impairments in older adults: National health and nutrition examination surveys 1999–2004. Nutr. Res..

[30] Lim JU, Lee JH, Kim JS, Hwang YI, Kim T-H, Lim SY, Yoo KH, Jung K-S, Kim YK, Rhee CK (2017). Comparison of World Health Organization and Asia-Pacific body mass index classifications in COPD patients. Int. J. Chron. Obstruct. Pulmon. Dis..

[31] Evans EM, Rowe DA, Racette SB, Ross KM, McAuley E (2006). Is the current BMI obesity classification appropriate for black and white postmenopausal women?. Int. J. Obes..

[32] Caring for Our Elders: Early Responses, India Ageing Report Available at: https://ruralindiaonline.org/en/library/resource/caring-for-our-elders-india-ageing-report-2017/. Accessed October 14 October 2022. (2017).

[33] National Family Health Survey (NFHS-5) INDIA Report | International Institute for Population Sciences (IIPS) Available at: https://iipsindia.ac.in/content/national-family-health-survey-nfhs-5-india-report. Accessed (June 29 June 2023).

[34] Han Z, Mulla S, Beyene J, Liao G, McDonald SD, Knowledge Synthesis Group (2011). Maternal underweight and the risk of preterm birth and low birth weight: A systematic review and meta-analyses. Int. J. Epidemiol..

[35] Pacific WHORO for the W The Asia-Pacific perspective : redefining obesity and its treatment. Sydney : Health Communications Australia Available at: https://iris.who.int/handle/10665/206936. Accessed March 16, 2024. (2000).

[36] James WPT, Chunming C, Inoue S (2002). Appropriate Asian body mass indices?. Obes. Rev..

[37] Dhoot J, Tariq S, Erande A, Amin A, Patel P, Malik S (2013). Effect of morbid obesity on in-hospital mortality and coronary revascularization outcomes after acute myocardial infarction in the United States. Am. J. Cardiol..

[38] Sandhu RK, Ezekowitz J, Andersson U, Alexander JH, Granger CB, Halvorsen S, Hanna M, Hijazi Z, Jansky P, Lopes RD, Wallentin L (2016). The ‘obesity paradox’ in atrial fibrillation: Observations from the ARISTOTLE (Apixaban for Reduction in Stroke and Other Thromboembolic Events in Atrial Fibrillation) trial. Eur. Heart J..

[39] Wienbergen H, Gitt AK, Juenger C, Schiele R, Heer T, Towae F, Gohlke H, Senges J, MITRA PLUS study group (2008). Impact of the body mass index on occurrence and outcome of acute ST-elevation myocardial infarction. Clin. Res. Cardiol..

[40] Diercks DB, Roe MT, Mulgund J, Pollack CV, Kirk JD, Gibler WB, Ohman EM, Smith SC, Boden WE, Peterson ED (2006). The obesity paradox in non–ST-segment elevation acute coronary syndromes: Results from the Can Rapid risk stratification of Unstable angina patients Suppress ADverse outcomes with Early implementation of the American College of Cardiology/American Heart Association Guidelines Quality Improvement Initiative. Am. Heart J..

[41] Flegal KM, Kit BK, Orpana H, Graubard BI (2013). Association of all-cause mortality with overweight and obesity using standard body mass index categories: A systematic review and meta-analysis. JAMA.

[42] Hjellvik V, Selmer R, Gjessing HK, Tverdal A, Vollset SE (2013). Body mass index, smoking, and risk of death between 40 and 70 years of age in a Norwegian cohort of 32,727 women and 33,475 men. Eur. J. Epidemiol..

[43] Mortality GBMI, Collaboration null Di Angelantonio E, Bhupathiraju S (2016). Body-mass index and all-cause mortality: Individual-participant-data meta-analysis of 239 prospective studies in four continents. Lancet.

[44] Bhaskaran K, Dos-Santos-Silva I, Leon DA, Douglas IJ, Smeeth L (2018). Association of BMI with overall and cause-specific mortality: A population-based cohort study of 3·6 million adults in the UK. Lancet Diabetes Endocrinol..

[45] Held C, Hadziosmanovic N, Aylward PE, Hagström E, Hochman JS, Stewart RAH, White HD, Wallentin L (2022). Body mass index and association with cardiovascular outcomes in patients with stable coronary heart Disease—A STABILITY substudy. J. Am. Heart Assoc..

[46] Cai X, Hu J, Wen W, Wang M, Zhu Q, Liu S, Yang W, Dang Y, Hong J, Li N (2022). Association between the geriatric nutritional risk index and the risk of stroke in elderly patients with hypertension: A longitudinal and cohort study. Front. Nutr..

[47] Raposeiras Roubín S, Abu Assi E, Cespón Fernandez M, Barreiro Pardal C, Lizancos Castro A, Parada JA, Pérez DD, Blanco Prieto S, Rossello X, Ibanez B, Íñiguez Romo A (2020). Prevalence and prognostic significance of malnutrition in patients with acute coronary syndrome. J. Am. Coll. Cardiol..

[48] Zhang H, Chai X, Li S, Zhang Z, Yuan L, Xie H, Zhou H, Wu X, Sheng Z, Liao E (2013). Age-related changes in body composition and their relationship with bone mineral density decreasing rates in central south Chinese postmenopausal women. Endocrine.

[49] Pi-Sunyer FX (2002). The medical risks of obesity. Obes. Surg..

[50] CDC Effects of Overweight and Obesity. Centers for Disease Control and Prevention. Available at: https://www.cdc.gov/healthyweight/effects/index.html. Accessed August 27, 2023. (2022).

[51] Menon GR, Singh L, Sharma P (2019). National Burden Estimates of healthy life lost in India, 2017: An analysis using direct mortality data and indirect disability data. Lancet Glob. Health.

[52] Longitudinal Ageing Study in India (LASI) | International Institute for Population Sciences (IIPS) Available at: https://www.iipsindia.ac.in/lasi. Accessed 6 January 2023.

[53] Lee SB, Oh JH, Park JH, Choi SP, Wee JH (2018). Differences in youngest-old, middle-old, and oldest-old patients who visit the emergency department. Clin. Exp. Emerg. Med..

[54] Cohen-Mansfield J, Shmotkin D, Blumstein Z, Shorek A, Eyal N, Hazan H, CALAS Team (2013). The old, old-old, and the oldest old: continuation or distinct categories? An examination of the relationship between age and changes in health, function, and wellbeing. Int J Aging Hum Dev.

[55] Alterovitz SSR, Mendelsohn GA (2013). Relationship goals of middle-aged, young-old, and old-old Internet daters: An analysis of online personal ads. J. Aging Stud..

[56] Wu Q, Gu D, Gu D, Dupre ME (2021). Oldest-Old Adults. Encyclopedia of Gerontology and Population Aging.

[57] OHCHR and older persons OHCHR. Available at: https://www.ohchr.org/en/older-persons. Accessed 4 March 2024.

[58] Rudnicka E, Napierała P, Podfigurna A, Męczekalski B, Smolarczyk R, Grymowicz M (2020). The World Health Organization (WHO) approach to healthy ageing. Maturitas.

[59] 9.3: Age Categories in Late Adulthood Social Sci LibreTexts. Available at: https://socialsci.libretexts.org/Bookshelves/Psychology/Developmental_Psychology/Lifespan_Development_-_A_Psychological_Perspective_2e_(Lally_and_Valentine-French)/09%3A_Late_Adulthood/9.03%3A_Age_Categories_in_Late_Adulthood. Accessed 4 March 2024. (2019).

[60] Seshan VE, Gönen M, Begg CB (2013). Comparing ROC curves derived from regression models. Stat. Med..

[61] Park D, Lee J-H, Han S (2017). Underweight: Another risk factor for cardiovascular disease?. Medicine.

[62] Cui R, Iso H, Toyoshima H, Date C, Yamamoto A, Kikuchi S, Kondo T, Watanabe Y, Koizumi A, Wada Y, Inaba Y, Tamakoshi A, JACC Study Group (2005). Body mass index and mortality from cardiovascular disease among Japanese men and women: the JACC study. Stroke.

[63] Hu F, Xu L, Zhou J, Zhang J, Gao Z, Hong Z (2020). Association between overweight, obesity and the prevalence of multimorbidity among the Elderly: Evidence from a cross-sectional analysis in Shandong, China. Int. J. Environ. Res. Public Health.

[64] Mokdad AH, Serdula MK, Dietz WH, Bowman BA, Marks JS, Koplan JP (1999). The spread of the obesity epidemic in the United States, 1991–1998. JAMA.

[65] Pan W-H, Yeh W-T, Chen H-J, Chuang S-Y, Chang H-Y, Chen L, Wahlqvist ML (2012). The U-shaped relationship between BMI and all-cause mortality contrasts with a progressive increase in medical expenditure: A prospective cohort study. Asia Pac. J. Clin. Nutr..

[66] Banack HR, Stokes A (2017). The ‘obesity paradox’ may not be a paradox at all. Int. J. Obes..

[67] Blew RM, Sardinha LB, Milliken LA, Teixeira PJ, Going SB, Ferreira DL, Harris MM, Houtkooper LB, Lohman TG (2002). Assessing the validity of body mass index standards in early postmenopausal women. Obes. Res..

[68] Halligan S, Altman DG, Mallett S (2015). Disadvantages of using the area under the receiver operating characteristic curve to assess imaging tests: A discussion and proposal for an alternative approach. Eur. Radiol..

[69] Maxim LD, Niebo R, Utell MJ (2014). Screening tests: a review with examples. Inhal. Toxicol..

[70] Borkan GA, Hults DE, Gerzof SG, Robbins AH, Silbert CK (1983). Age changes in body composition revealed by computed tomography. J. Gerontol..

[71] Garrow JS, Webster J (1985). Quetelet’s index (W/H2) as a measure of fatness. Int. J. Obes..

[72] Schwartz MB, Brownell KD (2004). Obesity and body image. Body Image.

[73] Teachman BA, Gapinski KD, Brownell KD, Rawlins M, Jeyaram S (2003). Demonstrations of implicit anti-fat bias: The impact of providing causal information and evoking empathy. Health Psychol..

[74] Tomiyama AJ, Carr D, Granberg EM, Major B, Robinson E, Sutin AR, Brewis A (2018). How and why weight stigma drives the obesity ‘epidemic’ and harms health. BMC Med..

[75] Schwartz MB, Chambliss HO, Brownell KD, Blair SN, Billington C (2003). Weight bias among health professionals specializing in obesity. Obes. Res..

[76] Major B, Tomiyama J, Hunger JM, Major B, Dovidio JF, Link BG (2018). The Negative and Bidirectional Effects of Weight Stigma on Health. The Oxford Handbook of Stigma, Discrimination, and Health.

[77] Tomiyama AJ, Finch LE, Belsky ACI, Buss J, Finley C, Schwartz MB, Daubenmier J (2015). Weight bias in 2001 versus 2013: Contradictory attitudes among obesity researchers and health professionals. Obesity.

[78] Doval N 20% of population to be elderly by 2050: HelpAge India report. mint. Available at: https://www.livemint.com/Politics/z6BacVOwf5SvmpD9P1BcaK/20-of-population-to-be-elderly-by-2050-HelpAge-India-repor.html. Accessed July 26 July 2023. (2025).

[79] Heath EM, Adams TD, Daines MM, Hunt SC (1998). Bioelectric impedance and hydrostatic weighing with and without head submersion in persons who are morbidly obese. J. Am. Diet Assoc..

[80] Wagner DR, Heyward VH (1999). Techniques of body composition assessment: A review of laboratory and field methods. Res. Q. Exerc. Sport..

[81] Lohman TG (1981). Skinfolds and body density and their relation to body fatness: A review. Hum. Biol..

[82] Brownlee J (2016) Parametric and Nonparametric Machine Learning Algorithms. MachineLearningMastery.com. Available at: https://machinelearningmastery.com/parametric-and-nonparametric-machine-learning-algorithms/. Accessed 27 July 2023. (2016).

[83] Yse DL The Complete Guide to Decision Trees. Medium. Available at: https://towardsdatascience.com/the-complete-guide-to-decision-trees-28a4e3c7be14. Accessed 27 July 2023.

[84] Hayes T, Usami S, Jacobucci R, McArdle JJ (2015). Using classification and regression trees (CART) and random forests to analyze attrition: Results from two simulations. Psychol. Aging.

