# Supplementary material for: Age-appropriate BMI cut-offs for malnutrition among older adults in India
Source: Sci Rep. 2024 Jul 2;14:15072. doi: 10.1038/s41598-024-63421-0 (PMC11219785; doi:10.1038/s41598-024-63421-0)
Supplement: Supplementary file 1 — Supplementary Information. [file 41598_2024_63421_MOESM1_ESM.docx]

**Figure 1: Sample decision tree**

**Child node to root node**

**Binary Split**

**Level 1**

**Level 2**

**Level 3**

**Figure 2: Decision tree for participants aged 60 to 74 years in training dataset (CV outcomes)**


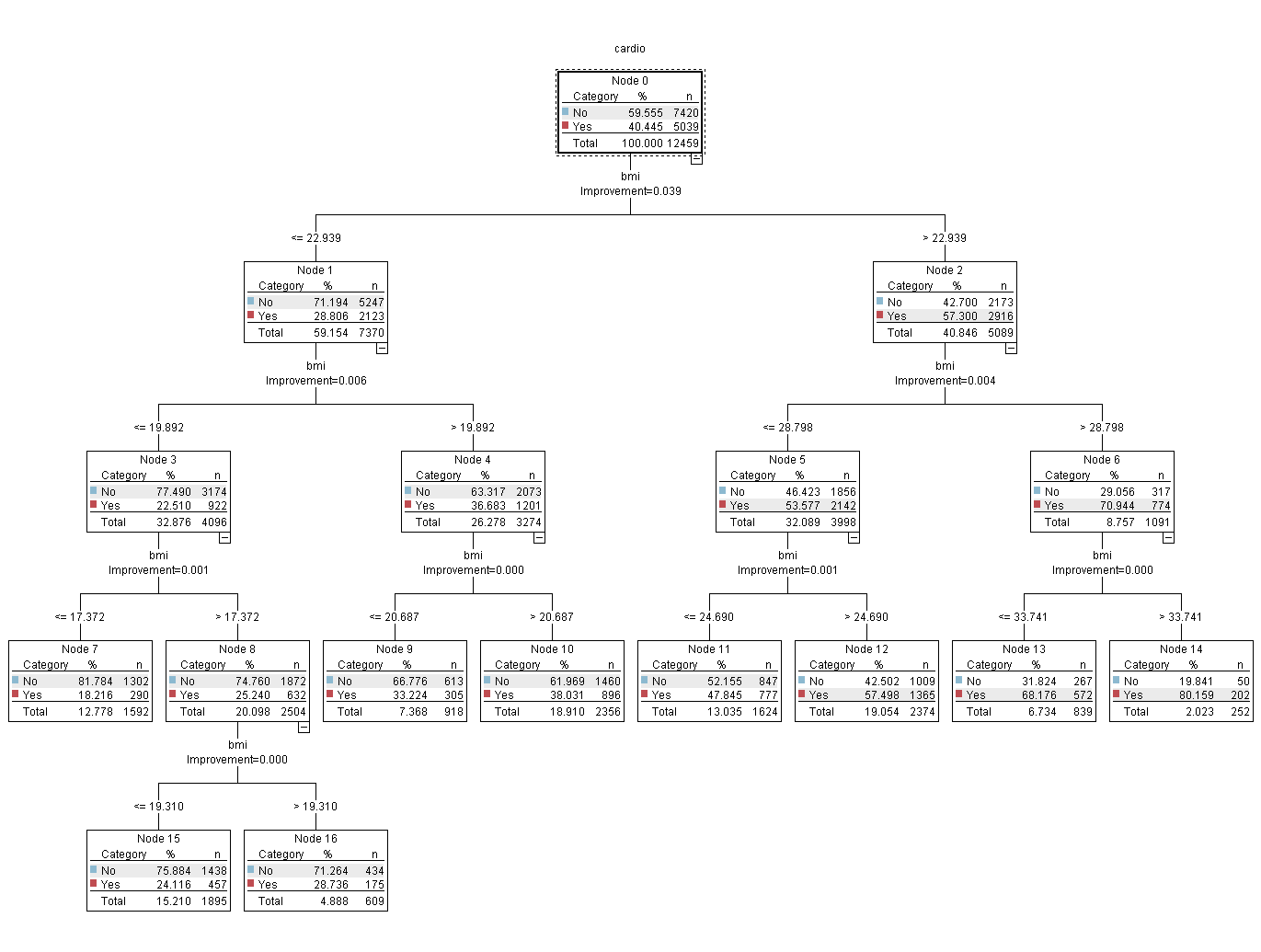


**Figure 3: Decision tree for participants aged 75 years and above in training dataset (CV outcomes) (N= 3981)**

**
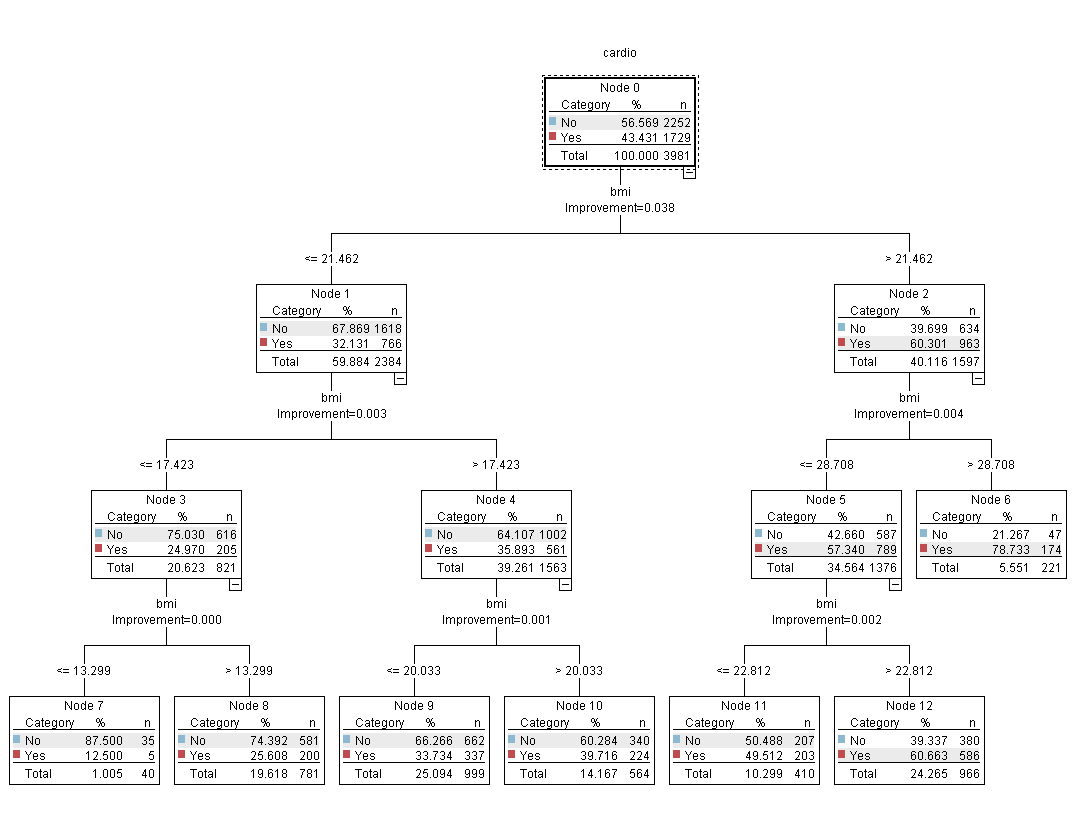
**

**Table 1: CART-Derived BMI Cut-Offs for Adults Aged 60 to 74 Years Old by CV Outcomes**

*Raw decision tree results for 60-74 years old subset of training dataset*

| Cut-offs | % with CV conditions (N) |
| --- | --- |
| <=17.4 | 18.2 (290) |
| >17.4 to <=19.3 | 24.1 (457) |
| >19.3 to <=19.9 | 28.7 (175) |
| >19.9 to <=20.7 | 33.2 (305) |
| >20.7 to <=22.9 | 38.0 (896) |
| >22.9 to <=24.7 | 47.9 (777) |
| >24.7 to <=28.8 | 57.5 (1365) |
| >28.8 to <=33.7 | 68.2 (572) |
| >33.7 | 80.2 (202) |

*Collapsed to:*

| Cut-offs | % with CV conditions (N) |
| --- | --- |
| <=17.4 | 18.2 (290) |
| >17.4 to<=19.9 | 30.5 (632) |
| >19.9 to <=22.9 | 36.7 (1201) |
| >22.9 to <=28.8 | 53.6 (2142) |
| >28.8 to <=33.7 | 68.2 (572) |
| >33.7 | 80.2 (202) |

**Table 2: CART-Derived BMI Cut-Offs for Adults Aged 75 Years and above by CV Outcomes**

*Raw decision tree results for 75 years and above subset of training dataset*

| Cut-offs | % with CV conditions (N) |
| --- | --- |
| <=13.3 | 12.5 (5) |
| >13.3 to <=17.4 | 25.6 (200) |
| >17.4 to <=20.0 | 33.7 (337) |
| >20.0 to <=21.5 | 39.7 (224) |
| >21.5 to <=22.8 | 49.5 (203) |
| >22.8 to <=28.7 | 60.7 (586) |
| >28.7 | 78.7 (174) |

*Collapsed to****:***

| Cut-offs | % with CV conditions (N) |
| --- | --- |
| <=13.3 | 12.5 (5) |
| >13.3 to <=20.0 | 30.2 (537) |
| >20.0 to <=21.5 | 39.7 (224) |
| >21.5 to <=22.8 | 49.5 (203) |
| >22.8 to <=28.7 | 60.7 (586) |
| >28.7 | 78.7 (174) |
